# Response to flutamide, as second-line therapy after bicalutamide, predicts efficacy of abiraterone, not that of enzalutamide

**DOI:** 10.1186/s13104-018-3453-z

**Published:** 2018-05-29

**Authors:** Yasushi Nakai, Nobumichi Tanaka, Makito Miyake, Takeshi Inoue, Satoshi Anai, Kiyohide Fujimoto

**Affiliations:** Department of Urology, Nara Medical University, 840 Shijo-cho, Kashihara, Nara 634-8522 Japan

**Keywords:** Castration-resistant prostate cancer, Hormonal therapy, Retrospective study

## Abstract

**Objective:**

The objective of this retrospective study was to evaluate whether the effect of second-line therapy of flutamide after bicalutamide can predict the response to abiraterone.

**Results:**

Thirty-four patients received abiraterone and 32 received enzalutamide after treatment with second-line flutamide for castration-resistant prostate cancer. Prostate-specific antigen-progression-free survival during treatment with abiraterone or enzalutamide was the endpoint. The response to flutamide therapy was defined as any decrease in prostate-specific antigen compared to baseline prostate-specific antigen. Among the abiraterone-treated patients, those for whom flutamide after bicalutamide was effective showed significantly lower prostate-specific antigen changes than those for whom it was ineffective (P = 0.0175). Prostate-specific antigen-progression-free survival was significantly higher in the abiraterone patients when flutamide was effective than in the patients when it was ineffective (P = 0.027). However, in enzalutamide-treated patients, the prostate-specific antigen changes were not significantly different between those for whom flutamide after bicalutamide was effective and those for whom it was ineffective (P = 0.75). In the enzalutamide patients, prostate-specific antigen-progression-free survival was not significantly different between those for whom flutamide was effective and those for whom it was ineffective (P = 0.92). Therefore, the response to second-line flutamide predicts the efficacy of abiraterone. This information should be helpful when choosing between abiraterone and enzalutamide for patients with castration-resistant prostate cancer.

## Introduction

Abiraterone was approved for castration-resistant prostate cancer (CRPC) at nearly the same time as enzalutamide was approved in both the USA and Japan. Abiraterone suppresses CRPC by inhibiting cytochrome P450 (CYP) 17 [1], while enzalutamide suppresses CRPC by acting as a selective antagonist of the androgen receptor (AR) [2, 3]. Although their mechanisms are different, the clinical efficacies of these two drugs seem very similar. Therefore, in clinical practice, deciding which agent should be used as first-line therapy for patients with CRPC has been problematic.

Considering the mechanism of abiraterone, Kim et al. [4] reported that the level of dehydroepiandrosterone (DHEA) was a good predictor of prostate-specific antigen (PSA) response in patients with CRPC who were treated with abiraterone, and that the level of DHEA in all patients was undetectable after 8 weeks of abiraterone treatment. In our previous randomized study, which compared the levels of DHEA in castration-sensitive prostate cancer patients who were treated with flutamide versus bicalutamide monotherapy, flutamide lowered DHEA levels, but bicalutamide did not [5]. Ayub et al. [6] reported that flutamide decreased the serum level of DHEA sulfate by inhibiting the activity of CYP17. The efficacy of flutamide as a second-line therapy after first-line bicalutamide has been reported; in this situation, second-line flutamide decreased PSA in 60% of patients with CRPC, with 34% of CRPC patients showing PSA decreases of at least 50% [7]. Narimoto et al. [8] showed that flutamide, as a second-line treatment after first-line bicalutamide, decreased the levels of DHEA, androstenedione, and androstenediol. These results indicate that second-line hormonal therapy using flutamide after bicalutamide decreases adrenal androgens and is effective for bicalutamide-refractory prostate cancer, although other mechanisms have been reported [9, 10]. Taking these results into consideration, the effect of second-line hormonal therapy using flutamide after bicalutamide should predict the effect of abiraterone. Therefore, we retrospectively evaluated this hypothesis.

## Main text

### Patients and methods

The study was conducted in accordance with the provisions of the Declaration of Helsinki, and the study protocol was approved by the ethics committee of Nara Medical University. Fifty-four patients received abiraterone and/or enzalutamide for CRPC between May 2014 and June 2017 at Nara Medical University Hospital. Forty-two of the 54 patients received flutamide as a second-line hormonal therapy for CRPC after bicalutamide, and these 42 patients were retrospectively evaluated. From these 42 patients, 34 received abiraterone and 32 received enzalutamide (Table 1). Abiraterone was used as the subsequent treatment after enzalutamide in 20 patients, and enzalutamide was used as the subsequent treatment after abiraterone in 4 patients. PSA progression during treatment with abiraterone or enzalutamide was defined as an increase in PSA value by > 25% relative to baseline or nadir PSA value after abiraterone or enzalutamide treatment [11]. The response to second-line hormonal therapy of flutamide was defined as any decrease in PSA value compared to baseline PSA before the second-line hormonal therapy. PSA progression-free survival during treatment with abiraterone or enzalutamide was the primary endpoint.

**Table 1:** Patient characteristics in this study

Median (range) or n (%)	Abiraterone (n = 34)	Enzalutamide (n = 32)	P-value
Age	75 (63–93)	75 (51–95)	0.52
Initial PSA	87 (6–10,800)	112 (6–10,800)	0.67
PSA prior to each agent	42 (3–3553)	13 (0.5–3043)	0.42
Gleason score			
≤ 6: 7: 8: ≥ 9: unknown	4:5:4:19:2	2:7:4:18:1	0.91
T stage			
≤ T2: T3: T4	24:8:2	24:6:2	0.89
N1	13 (38)	13 (40)	0.84
M1	18 (53)	17 (50)	0.98
Line			
1st: 2nd: 3rd: 4th: 5th	7:11:9:6:1	8:14:7:3:0	0.63
Chemotherapy history prior to each agent	11 (32)	10 (31)	0.92
Flutamide effective as second-line	15 (44)	15 (47)	0.82

### Statistical analysis

Statistical analysis was carried out with SPSS for Windows (version 20.0; IBM, Armonk, NY, USA). Mann–Whitney *U* tests were used to compare continuous variables and the Chi square test was used for categorical variables. PSA-progression-free survival rates were estimated using the Kaplan–Meier method. The log-rank test was used to compare the survival rates. A P value < 0.05 was considered statistically significant.

### Results

**Fig. 1:**
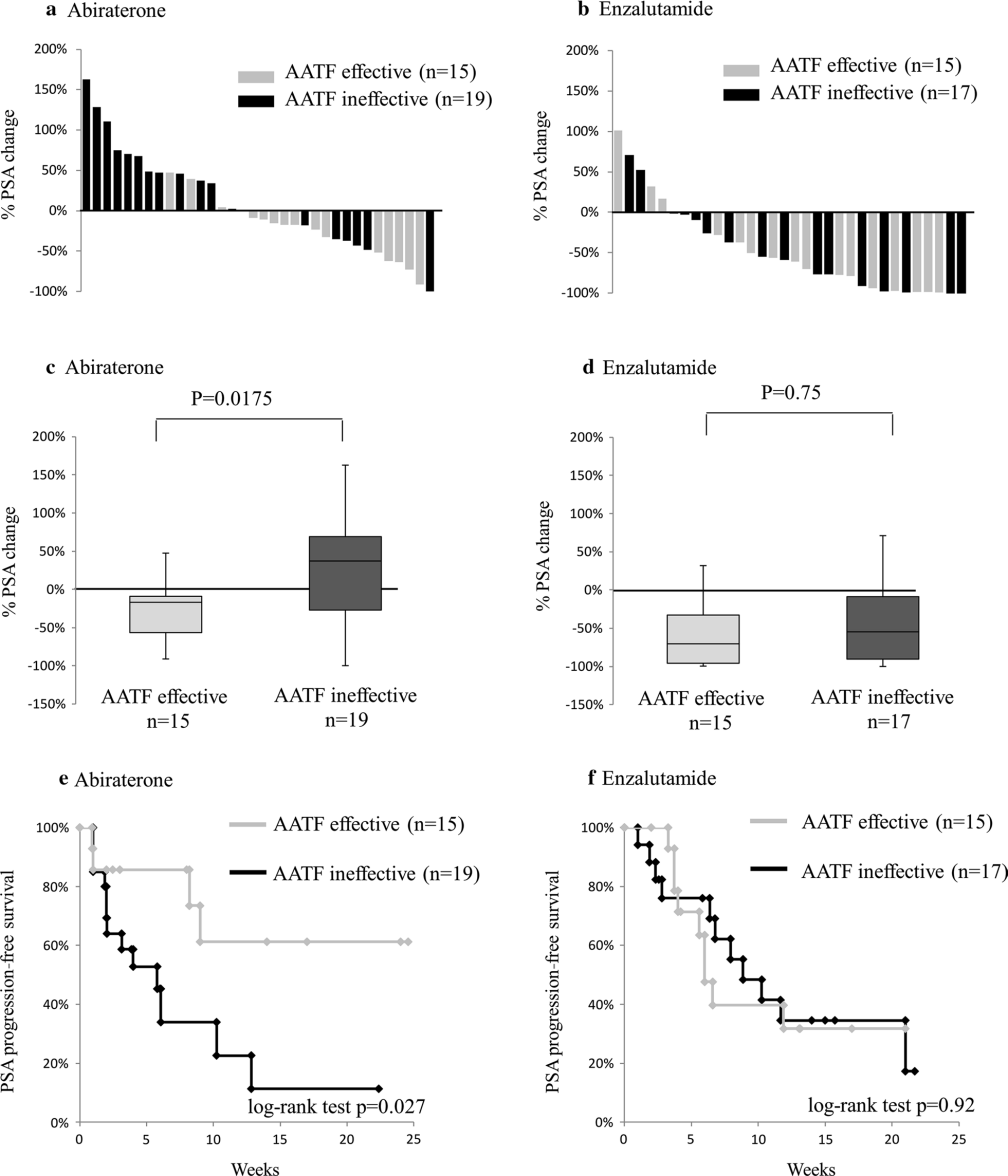
Responses of castration-resistant prostate cancer patients after second-line flutamide therapy. **a**, **b** Waterfall plots in patients treated with abiraterone (**a**) and enzalutamide (**b**). **c**, **d** Percent change in PSA in patients treated with abiraterone (**c**) and enzalutamide (**d**). **e**, **f** PSA-progression-free survival curves in patients treated with abiraterone (**e**) and enzalutamide (**f**). AATF, antiandrogen alternative therapy with flutamide

Waterfall plots of the patients treated with abiraterone and enzalutamide are shown in Fig. 1a, b, respectively, and the average percent changes in PSA after each treatment are shown in Fig. 1c, d, respectively. In patients treated with abiraterone, when flutamide after bicalutamide was effective, there was a significantly improved change in PSA compared to that when flutamide after bicalutamide was ineffective (Fig. 1c, P = 0.0175). In contrast, in patients treated with enzalutamide, there was no significant difference in the percent change in PSA between patients for whom flutamide after bicalutamide was effective and those for whom flutamide after bicalutamide was ineffective (Fig. 1d, P = 0.75).

In the abiraterone group, the PSA-progression-free survival in patients for whom flutamide after bicalutamide was effective (mean PSA-progression-free survival time: 17.3 months) was significantly greater (P = 0.027) than that in patients for whom flutamide after bicalutamide was ineffective (mean PSA-progression-free survival time: 7.2 months) (Fig. 1e). However, in the enzalutamide group, the PSA-progression-free survival curves were not significantly different (P = 0.92) between patients for whom flutamide after bicalutamide was effective (mean PSA-progression-free survival time: 10.6 months) and those for whom flutamide after bicalutamide was ineffective (mean PSA-progression-free survival time: 11.4 months) (Fig. 1f).

### Discussion

The agent (i.e., abiraterone or enzalutamide) that should be used first for patients with CRPC remains uncertain. Giving abiraterone after enzalutamide or enzalutamide after abiraterone has been evaluated in previous studies. Using retrospective analyses, Terada et al. [12] and Mori et al. [13] reported that treatment with abiraterone first followed by enzalutamide resulted in better progression-free survival than did treatment with enzalutamide first. However, a randomized trial of abiraterone after enzalutamide versus enzalutamide after abiraterone is ongoing [14], which should eventually provide more definitive answers. In the present study, there was no significant difference P = 0.55) in progression-free survival between abiraterone after enzalutamide (n = 20) and enzalutamide after abiraterone (n = 4) (data not shown). Given the current situation, another decision-making tool to help determine which agent should be used first for CRPC is needed.

Based on the present report, we conclude that the response to flutamide as a second-line therapy after bicalutamide predicts the response to abiraterone, which supports our hypothesis. A potential mechanism can be explained as follows. ① Abiraterone suppresses CRPC by inhibiting CYP17 [1]. ② The level of DHEA has been shown to negatively predict the response to abiraterone for patients with CRPC [4]. ③ Flutamide after bicalutamide can be effective in lowering the level of DHEA [5, 6, 8]. Therefore, the response to flutamide as a second-line therapy after bicalutamide can predict the response to abiraterone.

### Conclusion

The response to flutamide as a second-line therapy after bicalutamide predicts the response to abiraterone. This information should be important for medical practitioners when choosing between abiraterone and enzalutamide for patients with CRPC.

## Limitations

Our study has some limitations. First, the number of patients who were treated with abiraterone after enzalutamide was much larger than the number of patients who were treated with enzalutamide after abiraterone. Second, the sample size is small. Therefore, multivariate analysis could not be performed, and the possibility that unmeasured confounding variables influenced the outcome exists. Third, this is a retrospective study, which can suffer from selection bias when choosing treatment groups. Fourth, the treatments prior to abiraterone or enzalutamide varied. Therefore, unmeasured confounding factors may have influenced the present results.

## Abbreviations

CRPC: castration-resistant prostate cancer
AATF: antiandrogen alternative therapy with flutamide
PSA: prostate-specific antigen
CYP: cytochrome P450
AR: androgen receptor
DHEA: dehydroepiandrosterone
